# Endocannabinoid System Dysregulation from Acetaminophen Use May Lead to Autism Spectrum Disorder: Could Cannabinoid Treatment Be Efficacious?

**DOI:** 10.3390/molecules26071845

**Published:** 2021-03-25

**Authors:** Stephen Schultz, Georgianna G. Gould, Nicola Antonucci, Anna Lisa Brigida, Dario Siniscalco

**Affiliations:** 1 Department of Cellular and Integrative Physiology, Center for Biomedical Neuroscience, University of Texas (UT) Health Science Center San Antonio, San Antonio, TX 78229, USA; stevendri0629@gmail.com (S.S.); gouldg@uthscsa.edu (G.G.G.); 2 Biomedical Centre for Autism Research and Therapy, 70126 Bari, Italy; info@antonucci.eu; 3 Department of Precision Medicine, University of Campania, 80138 Naples, Italy; brigida.annalisa@gmail.com; 4 Department of Experimental Medicine, University of Campania, 80138 Naples, Italy

**Keywords:** autism, endocannabinoids, acetaminophen, endocannabinoid system

## Abstract

Persistent deficits in social communication and interaction, and restricted, repetitive patterns of behavior, interests or activities, are the core items characterizing autism spectrum disorder (ASD). Strong inflammation states have been reported to be associated with ASD. The endocannabinoid system (ECS) may be involved in ASD pathophysiology. This complex network of lipid signaling pathways comprises arachidonic acid and 2-arachidonoyl glycerol-derived compounds, their G-protein-coupled receptors (cannabinoid receptors CB1 and CB2) and the associated enzymes. Alterations of the ECS have been reported in both the brain and the immune system of ASD subjects. ASD children show low EC tone as indicated by low blood levels of endocannabinoids. Acetaminophen use has been reported to be associated with an increased risk of ASD. This drug can act through the ECS to produce analgesia. It may be that acetaminophen use in children increases the risk for ASD by interfering with the ECS.This mini-review article summarizes the current knowledge on this topic.

## 1. Introduction

Autism spectrum disorder (ASD) is a life-long disability that manifests in early childhood and continues throughout the lifespan, producing huge social and economic impacts to society [1]. ASD is defined in the *Diagnostic and Statistical Manual of Mental Disorders*, *5th edition* [1], which combines the previously separate categories of autistic disorder, Asperger’s syndrome, and pervasive developmental delay—not otherwise specified. ASD’s core symptoms are deficits in social interactions and communication, along with repetitive patterns of behavior. The core symptoms can range in severity along three defined levels indicating the spectrum of disability.ASD risk has been shown to be due to both genetic and environmental risk factors in approximately equal proportions [2]. Early in 2008, we showed that acetaminophen use may increase the risk for ASD [3]. Since then, others have also shown an increased risk for ASD when children are given acetaminophen or are exposed to acetaminophen prenatally [4,5]. It has been shown that acetaminophen acts through the endocannabinoid system (ECS) to produce analgesia [6]. We have demonstrated ECS dysregulation in ASD as shown by lower levels of cannabinoid receptor 2 (CB2) in peripheral blood mononuclear cells [7]. ECS dysregulation may be the method by which acetaminophen use increases the risk for ASD and may point the way to a potential treatment. There are currently no approved medications to treat the core symptoms of ASD.

In Section 2, we will provide an overview of the ECS. In Section 3, we will show evidence for ECS disruption in ASD and in animal models for ASD. Section 4 will provide chemical information for acetaminophen as well as mechanisms for its analgesic action. Section 5 will introduce animal models of ASD and show evidence that acetaminophen may change behavior in these animals through the ECS. In Section 6, we will show evidence that acetaminophen use may increase ASD risk. Currently used medical treatments for ASD will be discussed in Section 7. Section 8 will provide evidence that cannabinoids may be useful in treating ASD through the ECS. The discussion and summary of these topics will be in Section 9.

## 2. Overview of the Endocannabinoid System

**Figure 1:**
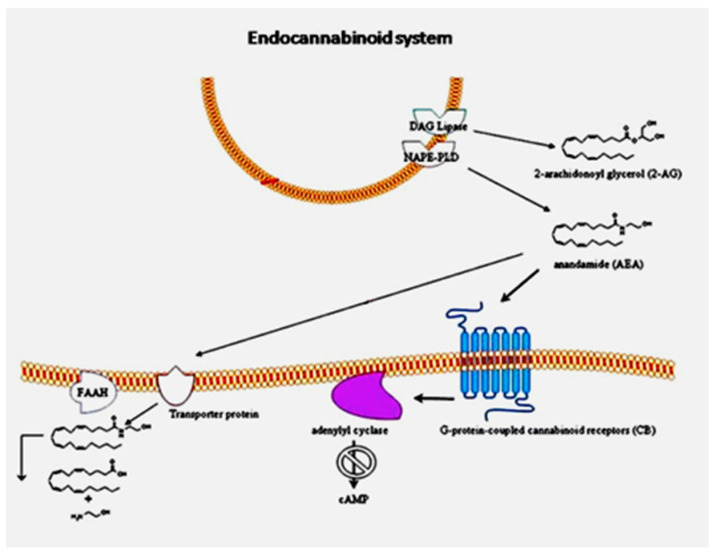
Endocannabinoid system workflow in peripheral blood mononuclear cells of patients with autism spectrum disorder (ASD). The cannabinoid receptor 2 (CB2) gene is over-expressed, as well as protein levels. The anandamide synthesis enzyme *N*-acyl phosphatidylethanolamine-specific phospholipase D (NAPE-PLD) gene is slightly decreased. Reprinted from [7], with permission from Springer (Berlin/Heidelberg, Germany) (license #5034221089815).

We have reviewed the link between the endocannabinoid system and ASD [8]. As shown in Figure 1 from our 2013 paper, the ECS consists of the classical cannabinoid receptors 1 and 2 (CB1 and CB2) along with the endocannabinoids that activate them, primarily anandamide and 2-arachidonylglycerol (2-AG), and the enzymes responsible for their synthesis and degradation. Diacylglycerol lipase (DAG lipase) is responsible for the synthesis of 2-AG, and *N*-acyl phosphatidylethanolamine-specific phospholipase D (NAPE-PLD) is the key enzyme for the synthesis of anandamide. The enzyme responsible for 2-AG degradation is monoacylglycerol lipase (MAGL), and the enzyme that degrades anandamide is fatty acid amide hydrolase (FAAH). The endocannabinoid receptors regulate cells by reducing the activation of adenylyl cyclase, thereby decreasing the production of cyclic adenosine monophosphate (cAMP). The ECS provides regulation for the immune system through CB2 receptors on immune system cells. CB1 receptors are found atthe highest concentration in the brain and the peripheral nervous systems, where they provide a regulatory function that is required for proper synaptogenesis, axon growth and positioning, and neuronal cell fate [9]. The lack of proper control over axon growth could result in improper development of the corpus callosum, which provides a pathway for axons traversing the brain from one hemisphere to the other.

## 3. Endocannabinoid System Disruption in ASD and ASD Animal Models

The BTBR T+tf/J (BTBR) inbred mouse strain was found to have an autism-like phenotype, and is used in ASD research. Wahlsten and colleagues (2003) have shown, in the BTBR mouse, that the corpus callosum is absent, which indicates a functional deficit in the brain’s inter-hemispheric communications [10]. A deficit has also been discovered in the corpus callosum of subjects with ASD. Wegiel and colleagues (2018) have shown that axon numbers, axon diameter, and axon area in the corpus callosum are reduced in the brains of subjects with ASD [11], indicating that the BTBR mouse strain is a good model for ASD. Defective corpus callosum development may be the result of a deficit in the ECS control of axonal growth and positioning. There are also signs of ECS disruption in the BTBR mouse strain.For example, CB1 density in the BTBR hippocampus is 15–20% higher, and CP55,940 (a cannabinoid, primarily CB1 receptor agonist [12])-stimulated GTPγS binding to Gi/o-coupled receptors is also elevated compared to other strains of mice, indicating a potential for increased sensitivity [13]. Furthermore, the treatment of BTBR mice with the FAAH inhibitor, URB597, ameliorated the characteristic social behavior deficits of BTBR mice [14]. Earlier studies showed that treatment of BTBR mice with the cannabinoid delta 9-tetrahydrocannabinol (Δ9-THC) increased spontaneous wheel running and enhanced active escape activities in the forced swim test [15]. There is also evidence for elevated CB2 receptor expression in the BTBR mouse brain [16].

**Figure 2:**
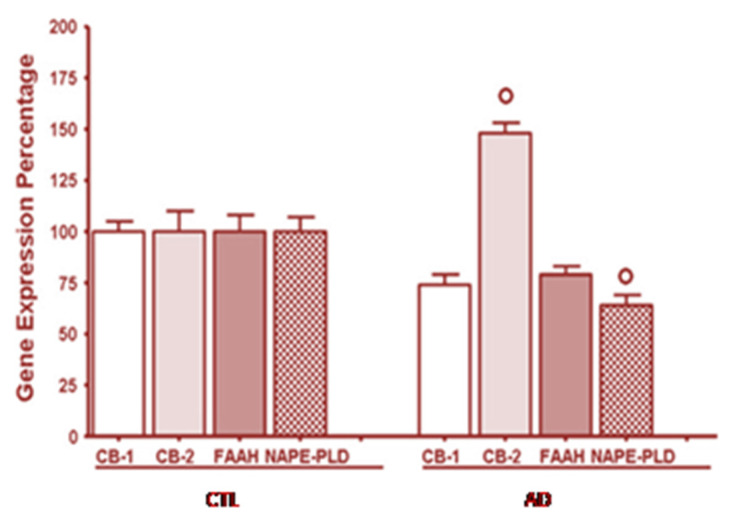
Over-expression of the CB2 receptor gene, but not of the cannabinoid receptor 1 (CB1) andfatty acid amide hydrolase (FAAH) enzyme, and down-expression ofthe NAPE-PLD gene in peripheral blood mononuclear cells of autistic children. CTL healthy control subjects, AD autistic patients. Reprinted from [7], with permission from Springer (license #5034221089815).

We have shown that the ECS is disrupted in children with ASD [7]. We investigated the involvement of the ECS in peripheral blood cells from children with autism compared to age-matched, normal, healthy, developing, control children, and we have shown the results from our study in Figure 2.

The mRNA level for cannabinoid receptor type 2 (CB2) was significantly increased compared to healthy subjects, whereas CB1 and FAAH mRNA levels were unchanged. The CB2 receptor regulates the immune response in these immune system cells and these results indicate immune system dysregulation. Also, the mRNA for *N*-acyl phosphatidylethanolamine phospholipase D (NAPE-PLD), the enzyme for anandamide synthesis, is significantly decreased in children with autism. This event would lead to less anandamide synthesis and indicates that ASD children should have decreased endocannabinoid tone, as defined by lower anandamide levels.

Our 2016 paper showed the relationship between acetaminophen use for fever and ASD. We suggested that endocannabinoid tone would be found to be lower in children with ASD and suggested that this would be a consequence of acetaminophen use [17]. Aran and colleagues (2018) have shown lower circulating endocannabinoid levels in children with ASD [18], thereby confirming our hypothesis that there would be lower endocannabinoid tone in individuals with ASD. They found that three endocannabinoids, anandamide (AEA), oleoylethanolamine (OEA), and palmitoylethanolamine (PEA), in the blood of children with ASD were significantly lower than in the controls.

We have reviewed EC signal dysregulation in ASD with emphasis on a correlation between inflammatory state and neuro-immune alterations [19]. Our paper shows that four pro-inflammatory cytokines, interleukin-1B, interleukin-6, interleukin-17, and tumor necrosis factor-alpha, are increased in the brains of individuals with ASD. This indicates an ongoing inflammatory process in the brain of these individuals. This inflammation may be responsible for the lack of proper brain development in individuals with ASD. Since the ECS provides regulation of immune system cells through CB2 receptors, disruption in this regulation could be responsible for the increased pro-inflammatory state in the ASD brain.

## 4. Chemistry of Acetaminophen and Mechanism of Analgesic Action

**Figure 3:**
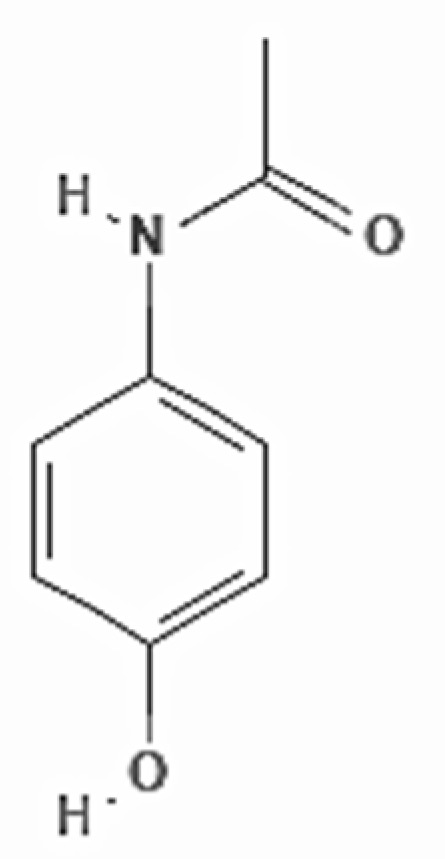
Chemical structure of acetaminophen, as adapted from Pubchem.

Acetaminophen (IUPAC name: *N*-(4-hydroxyphenyl)acetamide; CAS number: 103-90-2) is an amide with the chemical formula C_8_H_9_NO_2_, and a molecular weight of 151.16 g/mol (Figure 3). It is an odorless, bitter-tasting, white, solid crystal. It is soluble in alcohol and moderately soluble in water. It has a pH of about 6 in saturated aqueous solution, and the pKa is 9.38. It has a density of 1.3 g/cm^3^.

The exact mechanism of action as a pain reliever is still not fully understood. This drug shows weaker inhibitory effects on cyclooxygenase than those of other non-steroidal anti-inflammatory drugs [20]. Acetaminophen has the capacity to inhibit the prostaglandin G/H synthase-1 and -2 enzymes (also referred to as cyclooxygenase (COX)-1 and -2) in the central nervous system (CNS). These enzymes are able to catalyze the oxidization of arachidonic acid to prostaglandin G2, and subsequently catalyze the reduction of prostaglandin G2 to prostaglandin H2 [21]. Prostaglandins are responsible for facilitating nociceptive transmission in the CNS [22], and their decreased levels alleviate pain perception.

Interestingly, arachidonic acid is also a substrate for the biosynthesis of anandamide [23].

**Figure 4:**
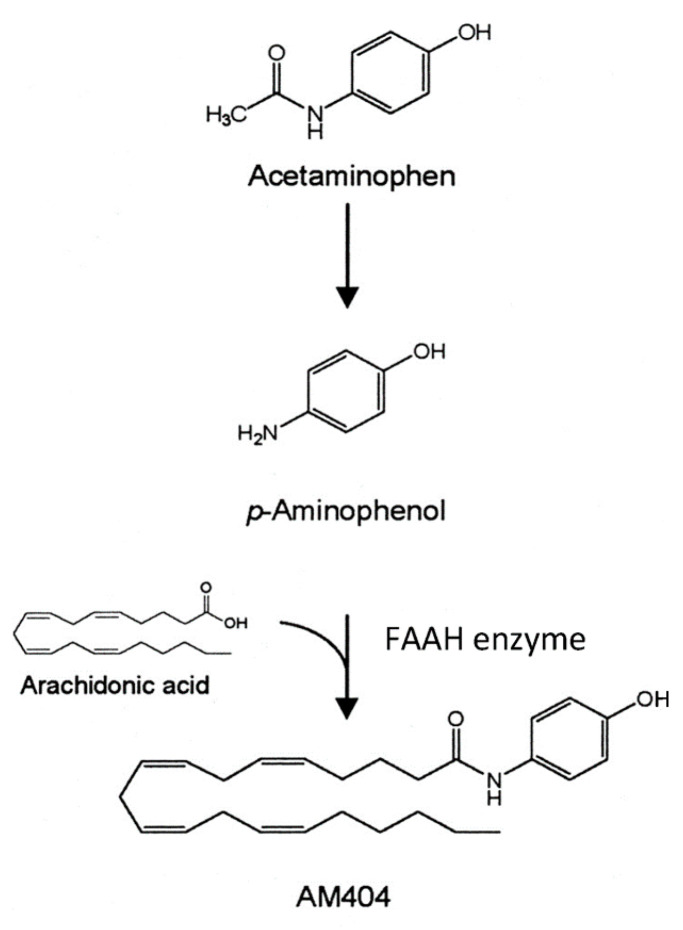
AM404 synthesis. Adapted from [6], under the terms of the Creative Commons CC-BY license.

A novel mechanism of action for acetaminophen to produce analgesia through the ECS has been described by Högestätt and colleagues [6]. They reported that acetaminophen produces analgesia through the activation of CB1 receptors, and that blocking these receptors eliminates the analgesic action of acetaminophen. Most acetaminophen (also called paracetamol) is metabolized in the liver through multiple mechanisms. However, in the CNS, the small amount of para-aminophenol produced from acetaminophen metabolism is combined with arachidonic acid to produce AM404 (Figure 4) [6]. This reaction is catalyzed by the action of the enzyme FAAH. FAAH is a central enzyme in the ECS where it catabolizes the two main endocannabinoids, anandamide and 2-arachidonoylglycerol. AM404 acts as an indirect agonist at the CB1 cannabinoid receptors by blocking the reuptake of the endocannabinoid, anandamide, thereby increasing its action in the ECS. This is a mechanism by which acetaminophen is thought to produce analgesia and may also disrupt the normal action of the ECS. We have hypothesized that in response to the repeated use of acetaminophen, the brain sets the level of anandamide lower, and these lower levels are what are seen in ASD as decreased endocannabinoid tone.

**Figure 5:**
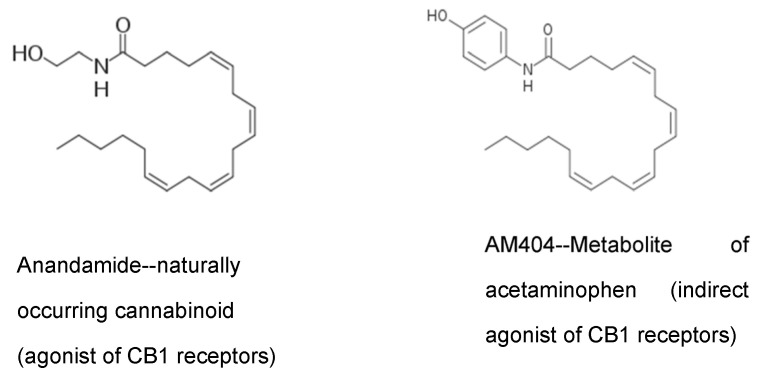
Chemical structure similarity between anandamide and acetaminophen metabolite AM404. Modified from https://commons.wikimedia.org/wiki/File:Anandamide_skeletal.svg (accessed on 20 February 2021), https://commons.wikimedia.org/wiki/File:AM404_skel.png (accessed on 20 February 2021), under the terms of CC BY-SA 3.0 license.

Note in Figure 5 the similarity in structure of the acetaminophen metabolite AM404 and anandamide, a naturally occurring endocannabinoid. AM404 is p-aminophenol conjugated with arachidonic acid. The similar structure of AM404 allows it to block the reuptake of anandamide from brain synapses. This effectively increases the amount of anandamide in the brain synapses.

## 5. Animal Models of Autism Spectrum Disorder Show the Effects of Acetaminophen on Behavior and Endocannabinoid Levels

**Figure 6:**
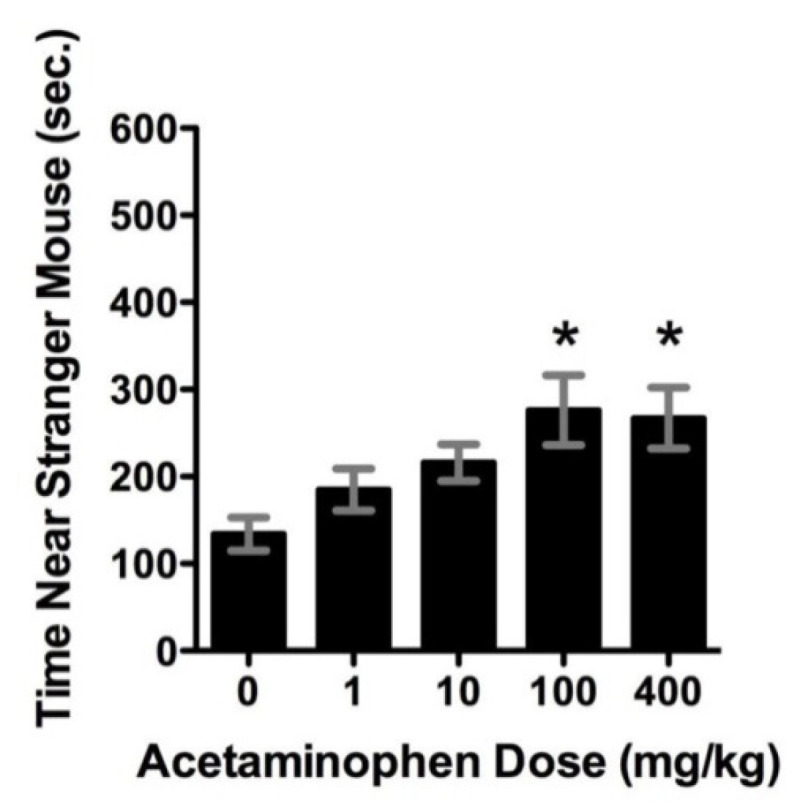
Acetaminophen treatment in an autism animal model. * indicates statistical significance (*p* < 0.05) versus non- treated animals. Reprinted from [24], with permission from Elsevier (Amsterdam, The Netherlands) (license #5034221282113).

Acetaminophen acts like a cannabinoid to improve sociability in the BTBR mouse model of autism [24]. Figure 6 shows the results of acetaminophen treatments in this mouse model of autism. The time investigating a stranger mouse is a test for sociability.This figure shows that acetaminophen significantly (*p* < 0.05) increased the time spent near a stranger for the two highest doses in the BTBR mouse model of autism.The improvement is similar to that achieved with buspirone, a serotonin agonist (data not shown). Acetaminophen and buspirone significantly increased, compared to controls, the time spent investigating stranger mice vs. novel objects.

Table 1 shows the effects of acetaminophen in the BTBR mouse model. This table shows that acetaminophen use along with social interaction tests can significantly increase the endocannabinoid anandamide in the brain frontal cortex of BTBR mice. The acetaminophen metabolite AM404 acts indirectly to increase levels of anandamide. This indirect agonist effect significantly (*p* < 0.05) increases anandamide in the frontal cortex similarly to that produced by social testing, as shown in Table 1. This increase in anandamide from acetaminophen is thought to be responsible for the increase in sociability, as shown in Figure 6.

In 2016, Servadio and colleagues confirmed the anandamide effect on behavior in a paper by using the valproic acid (VPA) rat model of ASD [25]. VPA dosing decreased rat sociability, using the three-chamber test. In this test, increasing anandamide in the brain by treating the rats with the FAAH inhibitor URB597 rescued the sociability of VPA-treated rats at post-natal day 35.

Kerr and colleagues also confirmed the effect of anandamide on sociability, again using the VPA rat model of autism. When the VPA-treated rats are given the FAAH inhibitor PF3845, anandamide is increased in the brain, which rescues social impairment [26]. In other words, increasing anandamide and endocannabinoid tone by inhibiting FAAH improves social behavior. It is also interesting that this effect is only seen in the males—this is further evidence that this is a good model for ASD, which is four times more prevalent in males.

## 6. Acetaminophen Use May Increase the Risk for ASD Which Could Be Due to Endocannabinoid System Disruption

In our 2008 paper, we demonstrated that acetaminophen, but not ibuprofen, use in children increases the risk for ASD [3]. Since our initial paper, additional authors have seen a similar risk for ASD from acetaminophen exposure in utero and early childhood [4,5]. As we have discussed, one way that acetaminophen produces analgesia is by stimulating cannabinoid receptors in the brain. Table 2 shows the association of analgesic use and autistic disorder (now known as ASD) from our 2008 paper.

In children aged 1–5 years, acetaminophen use after the measles–mumps–rubella vaccination significantly increased the odds of having ASD by more than six times. Ibuprofen use in these children was not significantly associated with autism.

**Figure 7:**
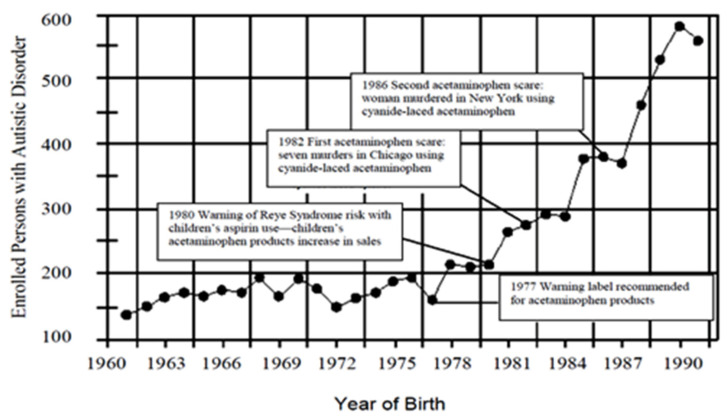
The number of persons with ASD by year of birth in California which was published by the state Department of Developmental Disabilities. Reprinted from [27], with permission from Elsevier (license #5034221427131).

Figure 7 is from our paper (Becker and Schultz, 2010) which shows an increase in autism cases by year in California, correlating to new stories about acetaminophen [27].

We have added several important events in the history of acetaminophen use in the US.

Note the following from this graph:

In 1980, the FDA warned of the possibility of Reye’s syndrome from aspirin use—acetaminophen use began increasing along with a precipitous increase in autism rates.

In 1982 and again in 1986, there were poisonings with cyanide-laced acetaminophen reported on the national news—note the decreases in individuals with those birth years, before the trend continues increasing.

The essential point we are making from this graph is that aspirin and acetaminophen have apparently affected the number of enrolled persons with autism in California. Bad news for aspirin, which presumably led to more acetaminophen use, and increased the number of children with autism. Reported bad news regarding acetaminophen, and presumably less acetaminophen use, decreased the number of children with autism with those years of birth.

In 2016, we published a study regarding fever in ASD using data from the National Database for Autism Research at the National Institutes of Health [17]. In this study, we again showed that acetaminophen use may be associated with autism (*p* = 0.013). Because these children are older than in our first study, the association is reversed; fewer children with ASD vs. non-ASD children use acetaminophen as a “first choice” compared to “never use” (*p* = 0.006). We also found that significantly more children with ASD vs. non-ASD children change to the use of ibuprofen when acetaminophen is not effective at reducing fever (*p* = 0.033), and we suggested that this change in use is due to ECS dysfunction from repeated use of acetaminophen, which would cause acetaminophen to lose effectiveness. We also found that children with ASD vs. non-ASD children are significantly more likely to show an increase in sociability when they have a fever (*p* = 0.037) and we hypothesized that this increase is due toa natural increase in anandamide from the fever, which would activate the ECS in ASD children with low endocannabinoid tone from early acetaminophen use.

Interestingly, very recently, it has been demonstrated that post-natal use of acetaminophen before the age of two is associated with an increase in the risk of ASD in male children [28], further confirming our previous results.

## 7. Current ASD Medical Treatments

There are currently no drugs approved by the Food and Drug Administration in the US for treatment of the core symptoms of ASD. There are two commonly prescribed atypical anti-psychotic drugs that are useful to treat ASD comorbidities, aripiprazole and risperidone. These drugs have been found to be relatively safe, and reduce irritability and self-injurious behavior. However, these drugs have side effects such as weight gain, sedation, dizziness, and nausea. Other drugs are often prescribed off-label for ASD co-morbidities, such as selective serotonin reuptake inhibitors, anti-psychotics, and anti-seizure drugs, but none of these drugs have proven to be a satisfactory treatment [29].

Vitamins, minerals, omega-3 fatty acids, and plant extracts have been tried as potential treatments but have only proved efficacious in a minority of patients. Stem cell treatments are currently being explored and have been shown to be safe in one clinical trial [30]. There is some hope that stem cells will decrease inflammation through the paracrine effect and return the brain to more normal functioning, but as of yet they are still in the experimental phase of development.

## 8. Cannabinoids May Treat ASD and Its Comorbidities

Since we have shown that the ECS appears to be disrupted in ASD, cannabinoids may be useful for ASD treatment. Cannabidiol (CBD) inhibits the action of the enzyme that metabolizes the anandamide, FAAH, and thereby increases the levels of anandamide. A brief report published by Aran and colleagues [18] showed the results of a parental survey of children with ASD. Parents reported that behavioral outbreaks were much improved or very much improved in 61% of patients. Evidence from the literature indicates that CBD alleviates many conditions co-occurring with ASD, such as self-injury and rage attacks, hyperactivity, sleep problems, and anxiety [31]; however, there has so far been no report indicating an improvement in autism core symptoms. In a placebo-controlled double-blind trial of three months’ duration in 150 ASD patients, two oral cannabinoid solutions demonstrated safety and good tolerability, but did not provide final evidence for efficacy [32].

The pharmacologic treatment with CBD may increase EC tone by increasing anandamide levels to improve the core symptoms as well.

Rett syndrome shares many of the features of ASD, including loss of language, stereotypic movements, and reduced cognition. Rett syndrome is caused by an X-linked dominant mutation in methyl-CpG-binding protein 2 (Mecp2). Mice containing the Mecp2 mutation are used as a model for this syndrome. Zamberletti and colleagues found that treatment of Mecp2 mice with the cannabinoid cannabidivarin (CBDV) seems to be effective in restoring memory deficits in these mice, as well as restoring normal CB1 and CB2 receptor levels in the brains of treated mice [33]. Indirect restoration of the ECS in mast and microglia cells treated with palmitoylethanolamide/luteolin has also been demonstrated in the VPA animal model of autism [34]. CBDV could also be tested in additional animal models of ASD and may prove to be an effective treatment to normalize the ECS. It may be that normalizing the ECS in individuals with ASD could be effective in treating their core symptoms as well as their co-morbidities.

## 9. Discussion

Acetaminophen is a commonly administered analgesic drug, and research has indicated that its use in children may increase the risk for autism. This risk may be due to a decrease in endocannabinoid tone in the brain. It may be possible to increase this tone through use of cannabinoids, such as CBD and CBDV, in order to increase endocannabinoid activity in the brain.

We have also shown that the MMR vaccine along with acetaminophen use may indirectly increase the risk for ASD [3]. This may be because acetaminophen use disrupts the normal functioning of the ECS to fight infection from the three vaccine viruses in the MMR vaccine. This would keep inflammatory cytokines at high levels in the brain and could disrupt the normal growth and myelinization of axons for neurons in the brain. We have reviewed the increase in inflammatory cytokines in the brain of individuals with ASD.

It has been shown that CBD improved some symptoms in individuals with ASD. It may be that the increase in endocannabinoid tone in ASD patients using CBD, or other drugswhich augment the amount of anandamide and other endocannabinoids in the brain, could be responsible for their improvement.

In summary, evidence for the association of acetaminophen use with increased risk of ASD has been presented. In ASD children, abnormal functioning of the ECS may be the result of acetaminophen use. Further research needs to be performed to evaluate if CBD, CBDV, or other cannabinoids, will be effective treatments for ASD.

## Figures and Tables

**Table 1:** Endogenous cannabinoid levels in the BTBR T+tf/J (BTBR) mouse frontal cortex. Reprinted from [24], with permission from Elsevier (license #5034221282113).

	No Behavior (70 min)		After SI/SN (70 min)	
Endocannabinoid	Saline	ACM	Saline	ACM
AEA (nmol/g)	11 ± 2	30 ± 7 *	26 ± 3 *	25 ± 3 *
2-AG (pmol/g)	3.8 ± 0.2	4.1 ± 0.3	4.6 ± 0.4	4.8 ± 0.4
OEA (pmol/g)	65 ± 11	43 ± 5	65 ± 2	58 ± 4

* Significantly different from controls (*p* < 0.05). *n* = 5–9 mice per group.

**Table 2:** Association of analgesic use and ASD. * indicates statistical significance (*p* < 0.05). Adapted from [3].

	Odds Ratio	95% CI	*p* Value
Children 1–18 years			
Acetaminophen	2.13	0.97–4.66	0.059
Ibuprofen	1.62	0.34–7.73	0.544
Children 1–5 years			
Acetaminophen	6.11	1.42–26.3	0.015 *
Ibuprofen	3.6	0.45–28.7	0.226
Children 1–18 years, cases limited to children with regression			
Acetaminophen	3.97	1.11–14.3	0.035 *
Ibuprofen	1.72	0.21–14.5	0.615
Children 1–18 years with post-vaccination sequelae			
Acetaminophen	8.23	1.56–43.3	0.013 *
Ibuprofen	0.89	0.10–8.30	0.918
